# Noncontrast MRI in assessing venous reflux of legs using QFlow analysis and radial basis function neural network technique

**DOI:** 10.1038/s41598-023-30437-x

**Published:** 2023-02-24

**Authors:** Min Yi Wong, Chien-Wei Chen, Yuan-Hsi Tseng, Shao-Kui Zhou, Yu-Hui Lin, Yao-Kuang Huang, Bor-Shyh Lin

**Affiliations:** 1 Division of Thoracic and Cardiovascular Surgery, Chiayi Chang Gung Memorial Hospital, Address: No. 6, W. Sec., Jiapu Rd., Puzi City, Chiayi County 61363 Taiwan (R.O.C.); 2 College of Photonics, National Yang Ming Chiao Tung University, Tainan City, 71150 Taiwan; 3 Department of Diagnostic Radiology, Chiayi Chang Gung Memorial Hospital, Puzi City, Chiayi County 61363 Taiwan; 4 College of Medicine, Chang Gung University, Taoyuan City 333, Taiwan; 5 Institute of Imaging and Biomedical Photonics, National Yang Ming Chiao Tung University, Address: No.301, Sec.2, Gaofa 3Rd Rd., Guiren Dist., Tainan City, 71150 Taiwan (R.O.C.)

**Keywords:** Diseases, Health care, Mathematics and computing

## Abstract

Since venous reflux is difficult to quantify, triggered angiography non-contrast-enhanced (TRANCE)-magnetic resonance imaging (MRI) is a novel tool for objectively evaluating venous diseases in the lower extremities without using contrast media. This study included 26 pre-intervention patients with superficial venous reflux in the lower extremities and 15 healthy volunteers. The quantitative flow (QFlow) analyzed the phase shift information from the pixels within the region of interest from MRI. The fast and simple radial basis function neural network (RBFNN) learning model is constructed by determining the parameters of the radial basis function and the weights of the neural network. The input parameters were the variables generated through QFlow, while the output variables were morbid limbs with venous reflux and normal limb classification. The stroke volume, forward flow volume, absolute stroke volume, mean flux, stroke distance, and mean velocity of greater saphenous veins from QFlow analysis could be used to discriminate the morbid limbs of pre-intervention patients and normal limbs of healthy controls. The neural network successfully classified the morbid and normal limbs with an accuracy of 90.24% in the training stage. The classification of venous reflux using the RBFNN model may assist physicians in clinical settings.

## Introduction

Lower extremity venous reflux, or venous insufficiency or incompetence, is a common venous hemodynamic disorder in which the venous blood flow bidirectionally returns to the heart due to primary dilation of the vein wall, valve destruction and primary valvular incompetence. Generally, the thin, flexible valve in all peripheral veins closes tightly to prevent backward blood flow; however, the destruction or damaging of the valve can lead to retrograde blood flow or the weakness of the vein wall results in vein dilation leading to valve cusps opening and eventual reflux^1,2^. Venous reflux may occur in the deep, superficial, or perforating venous systems of the lower extremities. The superficial venous system is the system that drains cutaneous microcirculation and is located between the skin and muscular fascia; it comprises the great saphenous vein (GSV), the small saphenous vein, anterior and posterior accessory saphenous veins, and numerous venous tributaries^3^. Superficial venous reflux of the lower extremities is a common medical problem associated with a wide range of clinical spectrum, from asymptomatic, cosmetic problems to severe symptoms, including spider veins, reticular veins, varicose veins, superficial thrombosis, pigmentation, venous ulceration, swollen limbs with deep vein thrombosis, and fatal outcomes, such as pulmonary emboli^4–6^.

Venous reflux has multiple long-term health effects; it affects the quality of life and causes an economic burden. Therefore, identifying this reflux in patients with venous disease is essential for treatment algorithms. Various noninvasive and invasive diagnostic tools are available to assess such patients. Duplex ultrasonography, which provides anatomical and gravitational reflux information, is usually the first-line diagnostic tool for assessing venous reflux^4,7^. In addition, computed tomography (CT) venography is reportedly less invasive than conventional angiography; however, the venous system is not evenly enhanced through CT venography, and high-quality enhancement requires specific access (a morbid limb). Most magnetic resonance venography techniques involving contrast media have exhibited good sensitivity in detecting lesions in vessels^8^. Compared to the first-line diagnostic tool Duplex ultrasonography and CT venography, triggered angiography non-contrast-enhanced (TRANCE)-magnetic resonance imaging (MRI) technique is effective (improves therapeutic decisions) and objective (same outcome for different users) analysis tool without using contrast agents.

The TRANCE technique produces a vascular image without contrast agents by recording differences in vascular signal intensities due to flow changes in the cardiac cycle for subsequent image subtraction^9^. TRANCE-magnetic resonance imaging (MRI) can reveal the locations of venous compressions and all major collateral veins and can thus achieve superior venous ablation results without requiring venipuncture^10,11^. We routinely apply this technique to evaluate the entire venous system in the lower extremities before interventions. In addition, quantitative flow (QFlow) technology is a new noninvasive quantitative method for identifying hemodynamic changes by calculating the flow-related parameters throughout the cardiac cycle. Since the MRI images are in pixel-by-pixel format, the quantitative analysis was performed by drawing the contour of the region of interest (ROI). QFlow analysis can quantitatively measure the hemodynamics of a region of interest according to the phase-shift information from each of the pixels. Artificial intelligence techniques are increasingly applied to improve the quality of healthcare services due to their ability to model large and complex medical information databases. The nonlinear modeling capabilities of artificial neural networks enable a powerful tool for clinical support decision systems and can easily be trained for identifying the patterns and extracting features using a small number of cases. Radial basis function neural network (RBFNN) has fast training speed and excellent approximation power, widely used in various fields.

In this study, we investigate hemodynamic patterns in patients with superficial venous reflux in the lower extremities through TRANCE-MRI and QFlow analysis and further classify the venous reflux patients in pre-intervention using significant markers obtained from QFlow through an RBFNN approach.

## Materials and methods

### Patients and clinical methods

The study was approved by the Institutional Review Board (IRB) of Chang Gung Memorial Hospital (No: IRB 202100938B0 and 202200062B0). Written informed consent was obtained from all participants involved in this study, and the study was performed in accordance with the approved guidelines. A total of 41 subjects (26 patients with superficial venous reflux in the lower extremities who were scheduled to undergo superficial venous interventions on their morbid limb and 15 healthy volunteers) were enrolled. The subjects underwent TRANCE-MRI for venous pathology in their lower extremities at a tertiary hospital between April 2017 and September 2021. Patients were excluded if they exhibited poor compliance or had comorbidities that prevented them from lying down for the 1-h TRANCE-MRI protocol. All patients received noninvasive color Doppler ultrasonography for the venous status in their lower limbs in the supine position under standard ultrasound scanning by a cardiologist before the scheduled TRANCE-MRI. The segmental QFlow hemodynamic and morphological examinations were further performed before intervention.

### MRI acquisition

The MRI was performed using a 1.5-Tesla MRI scanner (Philips Ingenia, Philips Healthcare, Best, The Netherlands) and a peripheral pulse unit trigger, with patients in the supine position. We evaluated all arterial system images by using a three-dimensional (3D) turbo spin-echo (TSE) technique during the systole and diastole periods. We performed TSE TRANCE imaging using the following parameters: repetition time (TR), 1 beat; echo time (TE), shortest; flip angle, 90°; voxel size, 1.7 × 1.7 × 3 mm^3^; and field of view (FOV), 350 × 420. The venous blood flow is moderately slow, leading the veins to appear bright throughout the cardiac cycle. By contrast, the arterial blood flow is relatively fast during the systole, causing signal dephasing and leading to flow voids. Accordingly, when systolic triggering is applied, the arteries appear black. During the diastole, the blood flow in the arteries slows; the signal is not dephased, and the arteries appear bright on diastolic scans. Subtracting the two-phased scans yields a 3D dataset of the arteries. Other images of the venous systems were evaluated through 3D TSE short-tau inversion recovery (STIR) during the systole periods. We performed TSE STIR TRANCE imaging using the following parameters: TR, 1 beat; TE, 85; inversion recovery delay time, 160; voxel size, 1.7 × 1.7 × 4 mm^3^; and FOV, 360 × 320. STIR provides additional background suppression due to its additional suppression of connective tissues. When systolic triggering is applied, the arteries appear black. The imaging process yielded a 3D dataset of the venous system, with no subtraction required.

**Figure 1:**
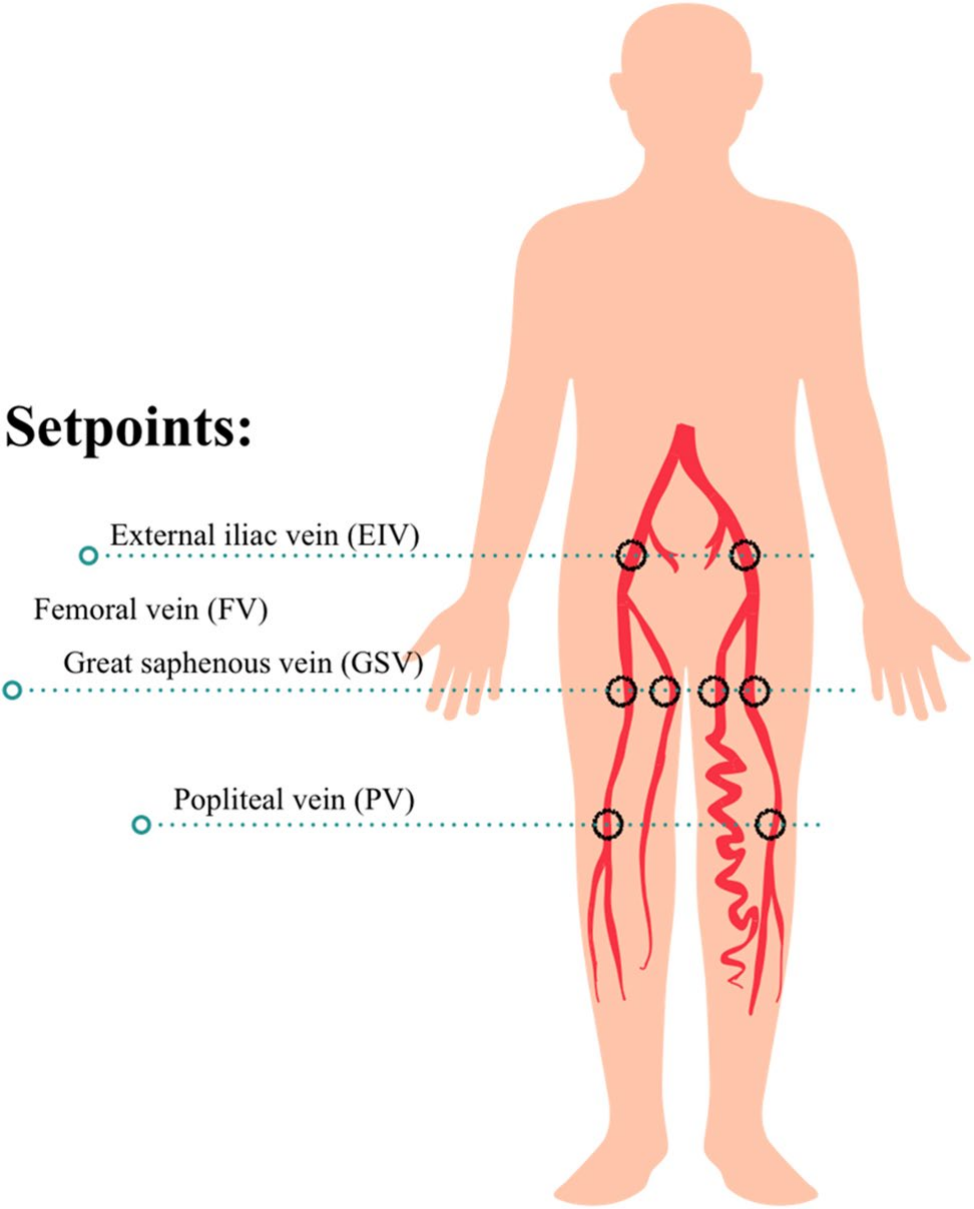
QFlow scan analysis of a region of interest at the setpoints. QFlow scanning is performed at four levels to obtain two-dimensional images of the vascular structure of the lower extremities containing phase-shift information. The ROI was drawn on the vascular lumens at the setpoints of the external iliac, femoral, greater saphenous, and popliteal veins to obtain hemodynamic variables for statistical analyses. Each setpoint of the QFlow can be used to generate eight flow-related variables.

We routinely performed QFlow scanning to determine the appropriate trigger delay times for systolic and diastolic triggering. All images were acquired without contrast media. The QFlow scan produced multiple acquisitions within one cardiac cycle (one R–R interval), resulting in multiple phases. QFlow scanning on a plane perpendicular to the blood flow was performed at four levels (abdominal and pelvic levels and arterial segments above the knee and segments below the knee) to obtain two-dimensional images containing phase-shift information of the vascular structure of the lower extremities (Fig. 1).

**Figure 2:**
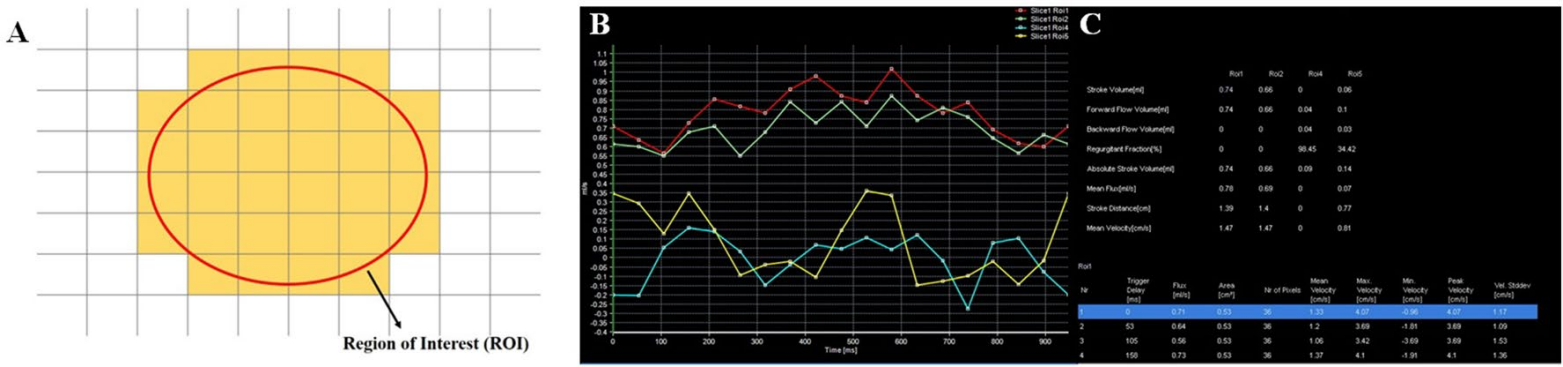
QFlow scanning through TRANCE-MRI. (**A**) Drawing Region of Interest (ROI) of pixel-by-pixel MRI image. (**B**) Two great saphenous veins and two popliteal veins with flow sequence by time. (**C**) QFlow parameters include stroke volume (SV), forward flow volume (FFV), mean flux (MF), stroke distance (SD), and mean velocity (MV) with different trigger delays.

The data set was quantitatively analyzed by drawing the contour of the ROI on the vascular lumens at the setpoints of the external iliac veins (EIVs), femoral veins (FVs), popliteal veins (PVs), and greater saphenous veins (GSVs) for every patient; the phase-shift information was analyzed from the pixels within the ROI (Fig. 2). Each setpoint of QFlow analysis can be used to generate eight flow-related variables, including stroke volume (SV), forward flow volume (FFV), backward flow volume (BFV), regurgitant fraction (RF), absolute stroke volume (ASV), mean flux (MF), stroke distance (SD), and mean velocity (MV). These parameters were used as objective indicators.

1. The SV (mL) is the net volume of blood passing through the contour of the ROI during one R–R interval.
2. The FFV (mL) is the volume of blood passing through the contour of the ROI in the positive direction (toward head direction) during one R–R interval.
3. The BFV (mL) is the volume of blood passing through the contour of the ROI in the negative direction (toward foot direction) during one R–R interval.
4. The RF (%) is the fraction of the backward flow to the forward flow.
5. The ASV (mL) is the absolute value of the forwarding flow volume plus the absolute value of the backward flow volume.
6. The MF (mL/s) is the stroke amount × heartbeat/60 (one R–R interval).
7. The SD (cm) is the net distance blood travels in the vessel during one R–R interval.
8. The MV (cm/s) is the SD × heartbeat/60 (one R–R interval).

### Radial basis function neural network

**Figure 3:**
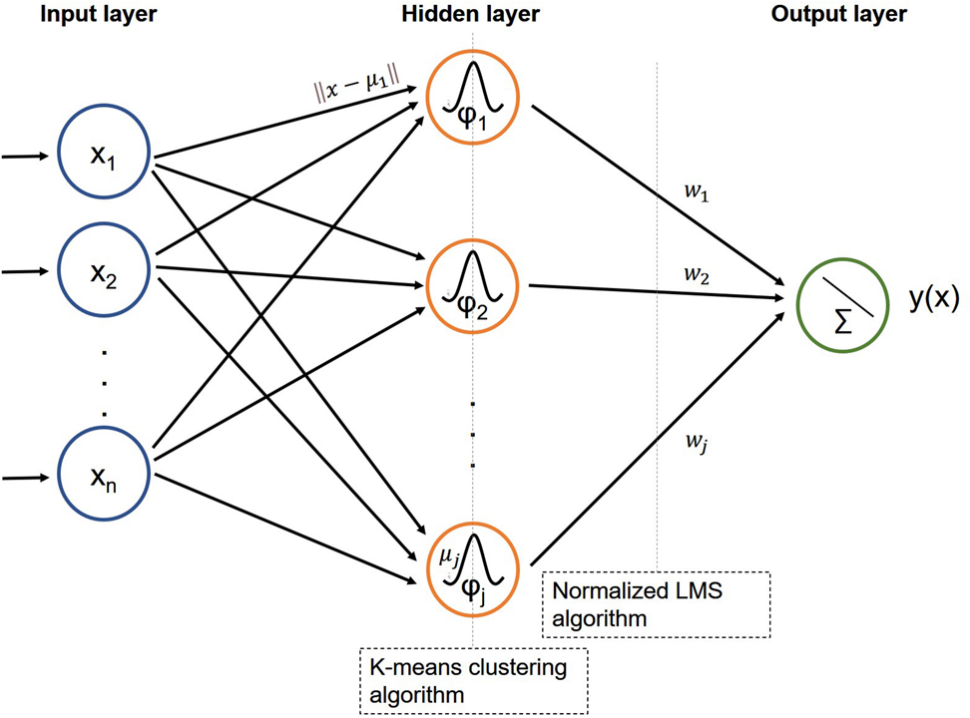
Schematic of the radial basis function neuron network.

A radial basis function neural network (RBFNN), the strengths of which include a simple network structure, excellent approximation capabilities, and fast training algorithms, was used. The RBFNN structure comprises three layers: an input layer, a single hidden layer, and an output layer, in which feedforward connections between the nodes form a single directed path rather than a loop (Fig. 3)^12,13^.

Each input vector $x$ is assigned to the neurons in the input layer and passes inputs to the hidden layers through weightless connections. Each hidden node in the hidden layer, computed using a Gaussian activation function, is a nonlinear function unit. The output ${\varphi }_{j}\left(x\right)$ of the jth hidden neuron obtained from the Gaussian functions with center ${\mu }_{j}$ is characterized by the following:

1 $${\varphi }_{j}\left(x\right)={\text{exp}}\left(-\frac{{\Vert x-{\mu }_{j}\Vert }^{2}}{2{\sigma }^{2}}\right),$$

where $\Vert x-{\mu }_{j}\Vert$ represents the Euclidean distance between input vector $x$ and center ${\mu }_{j}$, and $\sigma$ represents the standard deviation of the input vectors. The centers of the hidden neurons are trained using the k-means clustering approach, an unsupervised learning technique that centrally positions unit centers among training point clusters. The output $y(x)$ of the RBFNN model is connected to the hidden layer, with the linear combination of the weights trained using the normalized least mean square (LMS) algorithm and the Gaussian function ${\varphi }_{j}\left(x\right)$:

2 $${y}_{k}\left(x\right)=\sum_{j}{w}_{kj}{\varphi }_{j}\left(x\right).$$

In the training stage, the desired RBFNN outputs for patients with venous reflux and the healthy control groups are 1 and 0, respectively. After training, the RBFNN output is used as an index for differentiating patients as pre-intervention or healthy, with patients being considered pre-intervention if the output exceeds the threshold.

### Statistical analysis

An unpaired two-tailed Student’s *t* test was performed to analyze differences between pre-intervention patients with venous reflux and healthy individuals, with significance defined as *P* < 0.05. Statistical analyses were conducted using GraphPad Prism (version 5.0).

## Results

### Patient characteristics

**Figure 4:**
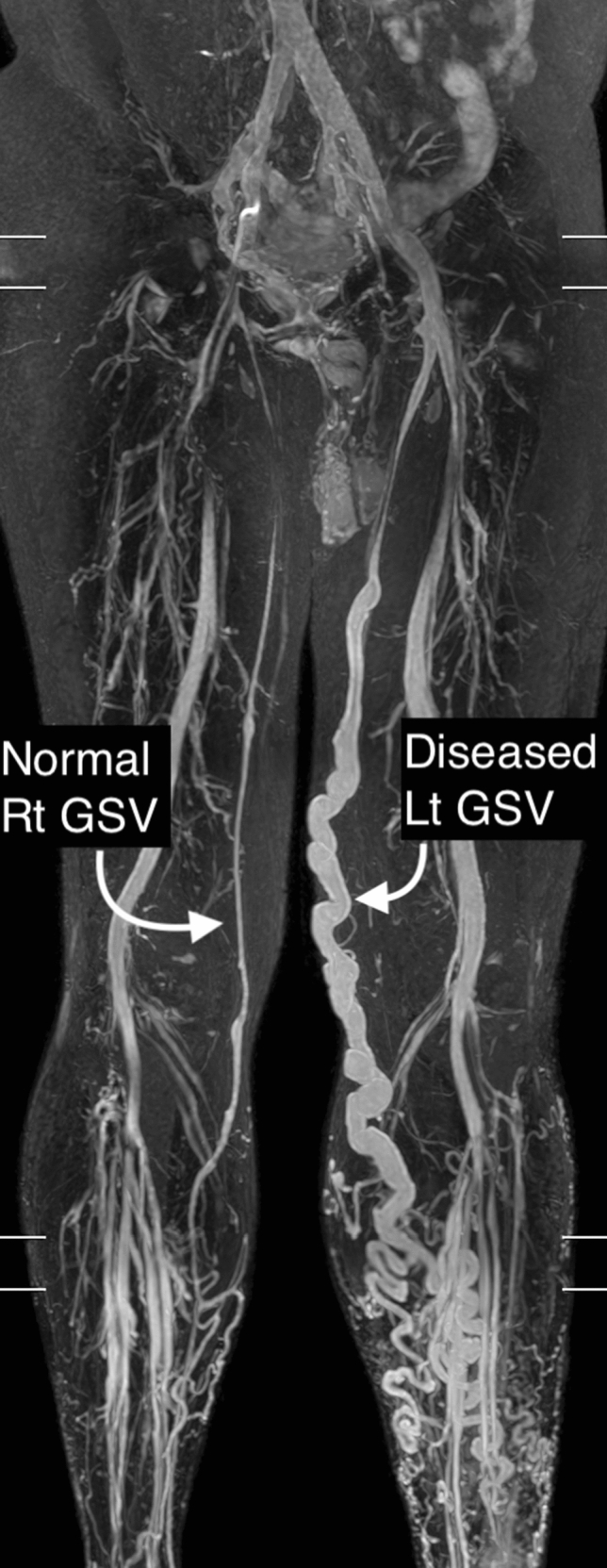
TRANCE-MRI image of a 68-year-old patient with venous reflux in the great saphenous vein of the left limb.

The descriptive characteristics, namely the sex, age, and comorbidities, of the 26 patients with venous reflux and the 15 healthy control individuals, are summarized in Table 1. The 26 patients with venous reflux had a mean age of 57.7 ± 11.6 years and were mostly women. The mean age of the healthy control was 27.1 ± 11.9 years and most were women. Regarding the use of TRANCE-MRI as the preoperative evaluation, 18 and 8 patients had venous reflux in the GSV of the left and right limbs, respectively. The TRANCE-MRI image of one patient with a diseased GSV in the left limb is presented in Fig. 4 as an example.

**Table 1:** Clinical characteristics of patients with venous reflux and the healthy control.

	Patients	Healthy control
Sex		
Male	6	2
Female	20	13
Age, year	57.7 ± 11.6	27.1 ± 11.9
BMI	27.19	23.6
Comorbidities		
Hypertension	4	0
Diabetes	2	1
CVA	1	0
Coronary artery disease	0	0
Deep vein thrombosis	0	0

### Performance of QFlow parameters

**Figure 5:**
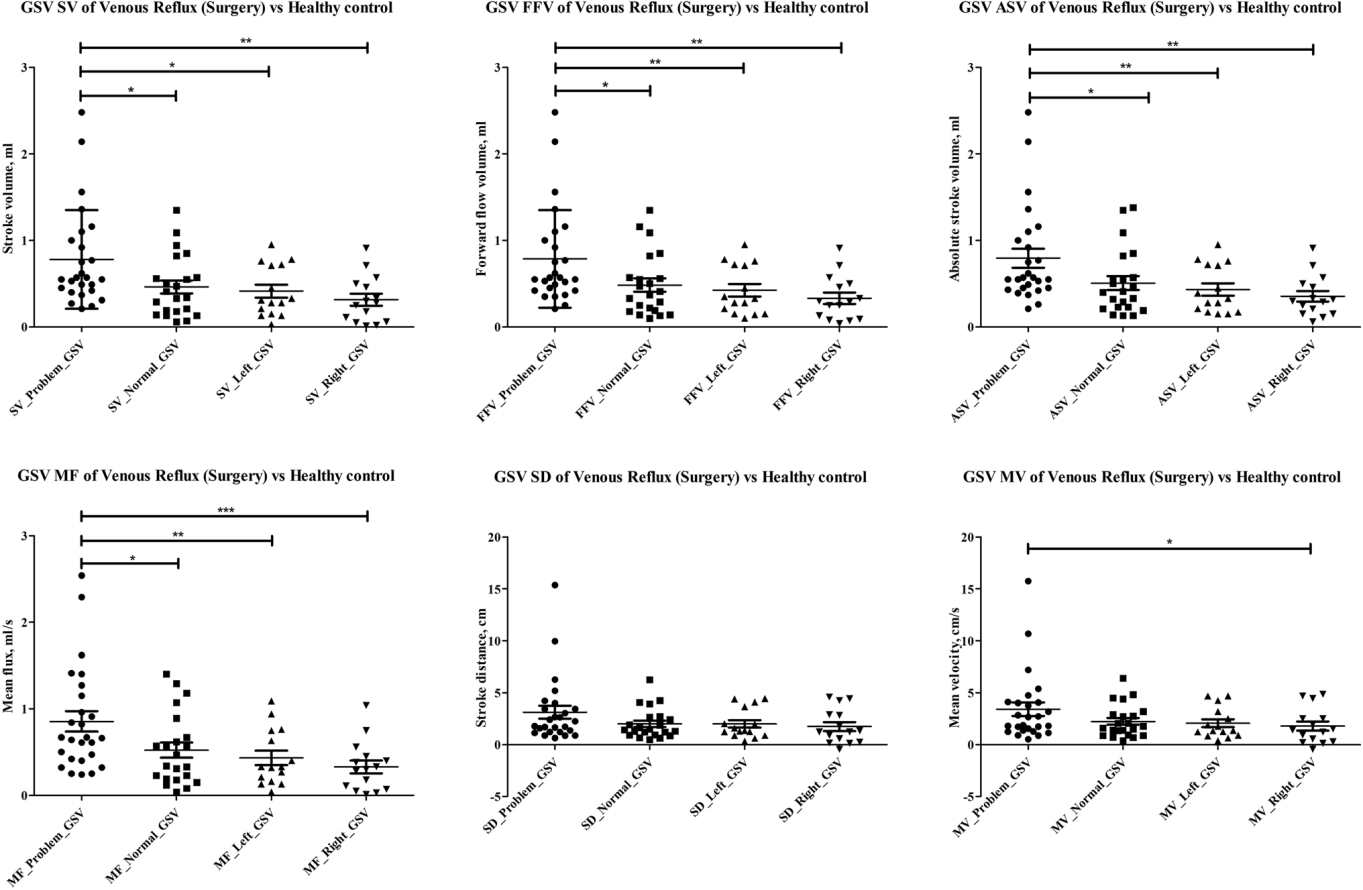
QFlow parameter comparison. Comparison of QFlow parameters (SV [mL], FFV [mL], ASV [mL], MF [mL/s], SD [cm], and MV [cm]) in the GSV segment for patients’ morbid and normal limbs and normal limbs of healthy controls; (*, *P* < 0.05; **, *P* < 0.01; ***, *P* < 0.001, unpaired two-tailed Student’s *t* test).

QFlow analysis through TRANCE-MRI was performed on 26 pre-intervention patients and 15 healthy controls by evaluating the SV (mL), FFV (mL), ASV (mL), MF (mL), SD (cm), and MV (cm) in EIV, FV, PV, and GSV segments. The performance of the QFlow parameters (SV, FFV, ASV, MF, SD, and MV) in discriminating pre-intervention patients and healthy controls was assessed by comparing patients’ morbid limbs with their normal limbs and the limbs of healthy controls in each venous segment. The SV, FFV, ASV, and MF in the GSV segment exhibited a significant ability to discriminate between morbid and normal limbs (patients and healthy controls), especially for right legs (Fig. 5). However, the QFlow parameters were unable to discriminate between morbid and normal limbs in the EIV, FV, and PV segments (data not presented).

### Classification of patients with venous reflux and healthy controls

RBFNN algorithms were implemented in the MATLAB programming environment (MATLAB 2020a). The performance of the RBFNN for the classification of pre-intervention patients with venous reflux and healthy controls was evaluated. According to our previous study, SD and MV in the GSV/PV ratio are able to discriminate reflux groups^6^. Therefore, the QFlow of the SV, FFV, ASV, and MF at the GSV and the GSV/PV ratio of the SD and MF were used as inputs. All 41 trials were used for training. The optimal threshold was determined in the training stage by using the F-measure Eq. (6), which was set from 0 to 1.

The performance of the proposed model was assessed according to three parameters: sensitivity, precision, and accuracy, which were evaluated from several binary classification parameters. The term true positive (TP) represents pre-intervention patients who were accurately diagnosed, and false negative (FN) represents pre-intervention patients inaccurately identified as healthy. Similarly, true negative (TN) represents healthy people who are accurately identified as such, and false positive (FP) represents healthy people who are inaccurately given a diagnosis.

Sensitivity (recall; TPR) refers to the ability to accurately identify actual pre-intervention patients.

3 $$\text{Sensitivity = }\frac{\text{TP}}{\text{(TP+FN)}}.$$

Precision (PPV) refers to the accuracy of pre-intervention identification.

4 $$\text{Precision = }\frac{\text{TP}}{\text{(TP+FP)}}.$$

Accuracy (ACC) refers to the correctness of identifying pre-intervention patients and healthy individuals.

5 $$\text{Accuracy = }\frac{\text{(TP+TN)}}{\text{(TP+TN+FP+FN)}}.$$

F-measure measures the model’s accuracy through the harmonic mean of the model’s precision and recall.

6 $$ {\text{F}} - {\text{measure}}\, = \,2\, \times \,\frac{{{\text{Recall}}\, \times \,{\text{Precision}}}}{{{\text{Recall}}\, + \,{\text{Precision}}}}. $$

**Figure 6:**
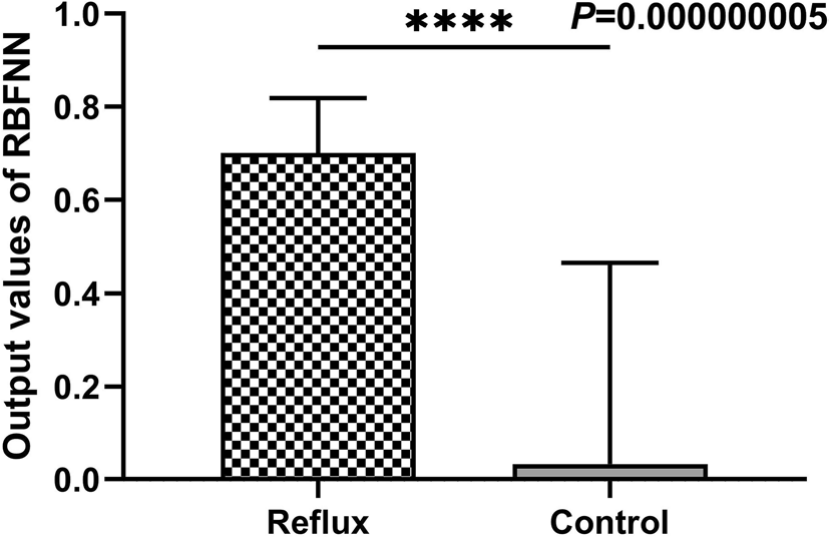
Correspondence of RBFNN outputs for the classification of pre-intervention patients with venous reflux and healthy controls. (****, *P* < 0.0001, unpaired two-tailed Student’s *t* test).

The most favorable TPR, ACC, PPV, and f-measure achieved with the training data were 100, 90.24, 92.31 and 92.31%, respectively, for 16 hidden neurons. Therefore, optimal performance was achieved when the threshold and the number of neurons in the hidden layer were set as 0.5 and 16, respectively. Figure 6 shows the correspondence output for classifying pre-intervention patients with venous reflux and healthy controls.

## Discussion

In this study, the TRANCE-MRI technique was performed to safely diagnose venous reflux before superficial venous interventions, such as venous truncal ablation, were commenced. Venous diseases, unlike arterial diseases, progress slowly with various manifestations, which often results in delayed diagnoses and unpredictable outcome. Patients suspected of having venous reflux disease of the legs undergo preoperative evaluations, such as air plethysmography and duplex ultrasonography (DUS), at the beginning of their therapy. DUS has been the primary choice for evaluating superficial venous insufficiencies because it enables hemodynamic information assessment and the site of the reflux to be anatomically defined^14,15^. DUS can be used to detect saphenous or perforating vein reflux and can be used for preoperative mapping. CT venography can be used for the preoperative evaluation of the venous system in the lower extremities, especially for deep venous thrombosis. However, this technique requires contrast medium injections and ionizing radiation that may be harmful to the morbid limb and cause adverse events, such as renal dysfunction and allergic reactions^16,17^.

TRANCE-MRI is a promising technique for imaging the veins and arteries of the entire lower extremities through an electrocardiography (ECG)-gated three-dimensional (3D) TSE sequence. This technique evaluates blood-flow velocity, and therefore, ECG gating is required to selectively obtain data during the desired cardiac phases through identifying signal differences between systolic-triggered and diastolic-triggered acquisitions^18^. Since 2017, our team has employed TRANCE-MRI to obtain valuable information for management of complex venous diseases^6,11,19–22^. Our capable radiological team could complete a full venous mapping within 20 min, under a reasonable cost (250 USD/each exam), thus TRANCE-MRI has become a standard preoperative evaluation for superficial venous interventions in our institutions and has positive feedback after preoperative communications.

According to the Edinburgh Vein Study in the United Kingdom^23^, the San Diego study in the United States^24^, and the Bonn Vein Study in Germany^25^, more women have superficial reflux than men, which is consistent with findings from the present study. Although venous reflux may occur in any part of the GSV, it particularly occurs at the origin of the GSV and in the lower third of the thigh, which are key segments^26^. Venous reflux develops when the valves above the saphenofemoral junction do not function properly, which in turn causes dilatation and valvular incompetence in the GSV and its tributaries^27^. TRANCE-MRI in pre-invention evaluations revealed the patients with venous reflux in this study to have diseased GSV morphologies and tributaries (Supplementary Video S1). We performed QFlow scans through TRANCE-MRI at the setpoints of EIV, proximal venous segments (FV and GSV), and distal venous segments (PV) of the lower extremities of patients and healthy controls. The QFlow parameters (SV, FFV, ASV, and MF) in the GSV segments exhibited a significant ability to discriminate between morbid and normal limbs, which is a key aspect of superficial venous reflux. To assess the effectiveness of the TRANCE-MRI and QFlow analysis in the superficial venous reflux, we classified the morbid limbs of pre-intervention patients and the normal limbs of healthy controls using an artificial neuron network, the RBFNN. The configuration of the neural network was performed using the results of a statistical experimental study to improve the predictive power of the network on an unseen data set. The neural network successfully classified morbid and normal limbs with an ACC of 90.24% and a PPV of 92.31% in the training stage. However, the small sample size may have caused bias in the classifications.

## Conclusion

TRANCE-MRI is a promising tool that facilitates vascular imaging of the lower extremity veins and can improve superficial venous intervention strategies for the lower extremities. QFlow through TRANCE-MRI can be used to verify the presence of superficial venous reflux in the lower extremities; the SV, FFV, ASV, and MF QFlow parameters in the GSV segment can be used to discriminate morbid from normal limbs. The classification of patients with venous reflux and healthy controls using RBFNN with QFlow parameters as input variables may aid in decision-making to assist the physician in venous reflux classification.

In this work, the major limitations are its non-randomized study with the limited patients’ number enrolled. Secondly, only patients with simple venous reflux were included. Future studies may potentially develop different artificial neural network models toward each type of venous diseases.

## Supplementary Information

Supplementary Video S1.

## Data Availability

The datasets generated during and/or analyzed during the current study are available from the corresponding author on reasonable request.
